# Mitochondriome and Cholangiocellular Carcinoma

**DOI:** 10.1371/journal.pone.0104694

**Published:** 2014-08-19

**Authors:** Wesam Bahitham, Xiaoping Liao, Fred Peng, Fiona Bamforth, Alicia Chan, Andrew Mason, Bradley Stone, Paul Stothard, Consolato Sergi

**Affiliations:** 1 Department of Laboratory Medicine and Pathology, University of Alberta, Edmonton, Alberta, Canada; 2 Department of Agricultural, Food, and Nutritional Science, University of Alberta, Edmonton, Alberta, Canada; 3 Department of Genetics, University of Alberta, Edmonton, Alberta, Canada; 4 Department of Medicine, University of Alberta, Edmonton, Alberta, Canada; 5 Department of Pediatrics, University of Alberta, Edmonton, Alberta, Canada; 6 Benaroya Research Institute, Seattle, Washington, United States of America; University of Palermo, Italy

## Abstract

Cholangiocellular carcinoma (CCA) of the liver was the target of more interest, recently, due mainly to its increased incidence and possible association to new environmental factors. Somatic mitochondrial DNA (mtDNA) mutations have been found in several cancers. Some of these malignancies contain changes of mtDNA, which are not or, very rarely, found in the mtDNA databases. In terms of evolutionary genetics and oncology, these data are extremely interesting and may be considered a sign of poor fitness, which may conduct in some way to different cellular processes, including carcinogenesis. MitoChip analysis is a strong tool for investigations in experimental oncology and was carried out on three CCA cell lines (HuCCT1, Huh-28 and OZ) with different outcome in human and a Papova-immortalized normal hepatocyte cell line (THLE-3). Real time quantitative PCR, western blot analysis, transmission electron microscopy, confocal laser microscopy, and metabolic assays including L-Lactate and NAD^+^/NADH assays were meticulously used to identify mtDNA copy number, oxidative phosphorylation (OXPHOS) content, ultrastructural morphology, mitochondrial membrane potential (ΔΨ_m_), and differential composition of metabolites, respectively. Among 102 mtDNA changes observed in the CCA cell lines, 28 were non-synonymous coding region alterations resulting in an amino acid change. Thirty-eight were synonymous and 30 involved ribosomal RNA (rRNA) and transfer RNA (tRNA) regions. We found three new heteroplasmic mutations in two CCA cell lines (HuCCT1 and Huh-28). Interestingly, mtDNA copy number was decreased in all three CCA cell lines, while complexes I and III were decreased with depolarization of mitochondria. L-Lactate and NAD+/NADH assays were increased in all three CCA cell lines. MtDNA alterations seem to be a common event in CCA. This is the first study using MitoChip analysis with comprehensive metabolic studies in CCA cell lines potentially creating a platform for future studies on the interactions between normal and neoplastic cells.

## Introduction

Cholangiocellular carcinoma (CCA), a primary malignancy of the biliary tract (intra- and extrahepatic), is the second most common hepatic cancer after hepatocellular carcinoma (HCC), the hepatocytic-based epithelial malignancy of the liver. Although there have been a number of investigations on CCA in the past, its carcinogenesis remains poorly understood. Remarkably, new environmental factors have been linked to its increased incidence in some geographic areas and CCA has been observed up to 20% as underlying malignancy of individuals who died for liver cancer in some studies [1]–[2]. CCA is a highly infiltrative tumor that expands and, usually, metastasizes within the liver and can be treated through surgical resection or liver transplantation if detected at an early stage, but most patients are diagnosed at an advanced stage [1]–[3]. Recently, the mitochondriome has been a fascinating focus of oncologic investigation, and somatic mitochondrial DNA mutations have been identified in some solid tumors suggesting a critical role in carcinogenesis [4]. Not only are mitochondria considered the powerhouse of the cell, but they also play an essential role in apoptosis. It has also been suggested that some molecular changes in mitochondrial DNA may shed some light on oncologic research. Mutations in mtDNA are enhanced by reactive oxygen species (ROS) generated by the oxidative phosphorylation pathway. Consequently, mtDNA seems to be more vulnerable to damage from ROS, because it is neither coated by chromatin nor associated with histones [5]. MtDNA mutations have been demonstrated in multiple types of human cancers, including hepatocellular carcinoma (HCC), breast cancer, ovarian cancer, and gastric cancer [4]. Some of these malignancies contain changes of mtDNA, which are not or, very rarely, found in the mtDNA databases. In terms of evolutionary genetics and oncology, this data are extremely interesting and may be considered a sign of poor fitness, which may conduct in some way to a number of different cellular processes, including carcinogenesis.

The mitochondrial genome is 16.6 kilobases (kb) long and contains 37 genes, which encode 13 proteins of the respiratory chain complexes, including seven, zero, one, three and two subunits of the complexes I through V respectively, 22 transfer RNAs and 2 ribosomal RNAs for the mitochondrial translational apparatus [6]. Mutations of mtDNA are present in coding and non-coding regions as well as in the D-loop, which is involved in the transcription and replication of mtDNA. Mutations in mitochondrial tRNA (mt-tRNA) genes are also recognized as a common cause of mitochondrial disorders [7]. Mutations in the D-loop region have been found in a variety of solid tumors [8]. However, the way in which D-loop mutations contribute to carcinogenesis remains unclear [9]. The molecular detection of cancer has been facilitated by a sensitive oligonucleotide-sequencing array that is known as the MitoChip. Studies involving the previous version of the MitoChip (v1.0) have attested its accuracy, which is validated by high-throughput sequencing of the mitochondrial genome. In fact, Zhou et al., using the MitoChip with an oligonucleotide-sequencing array, demonstrated a high incidence of mtDNA mutations in squamous cell carcinomas of the head and neck that may contribute to the development of a malignant phenotype by direct genotoxic effects from increased ROS production [10]. Similarly, Sui et al. conducted a related study and demonstrated the sensitivity of MitoChip by identifying mitochondrial mutations in premalignant gastrointestinal tract lesions [11].

Because the etiology of CCA remains obscure, mitochondriome investigations on this cancer may be valuable. Although extrahepatic CCA represents the majority of CCA cases, intrahepatic CCA subtype harbors worse prognosis than extrahepatic CCA. Thus, we focused on CCA considering the different outcome observed from the patients’ cancers from which the cells have been cultured and considering the prognostic implications of intrahepatic CCA. To our knowledge, this is the first report that contains a complete mitochondrial genome-sequencing array in CCA and represents the first investigation identifying the exact frequency of mitochondrial mutations utilizing a reproducible, array-based sequencing technology singularly coupled with metabolic assays.

## Materials and Methods

### Cell lines

CCA cell lines, HuCCT1, HuH28, and OZ, were obtained from the cell culture bank of the Japan Health Sciences Foundation in Tokyo and have been previously published in the literature [12], [13]. These cell lines were grown as a monolayer culture in their appropriate media. Human CCA cell lines including OZ and HuH-28 were derived from patients with CCA showing both extra- and intrahepatic involvement. HuCCT1 was derived from another CCA cell line arising from a malignancy in a patient with an intrahepatic CCA only. Briefly, HuCCT1 and HuH28 (cell lines from a moderately invasive CCA) were cultured in RPMI 1680 medium, Roswell Park Memorial Institute, (Invitrogen Canada Inc. Burlington, ON, Canada) supplemented with 10% Fetal Bovine Serum (FBS, PAA laboratories Inc. Etobicoke, ON, Canada), 1ml gentamicin, and incubated in a 5% CO_2_ chamber at 37°C. OZ (metastasizing cell lines) was grown in William E medium (Invitrogen Canada Inc. Burlington, ON, Canada) supplemented with 10% FBS. We included two HCC cell lines (HepG2, Huh-7) to our investigational study as well. Both cell lines were purchased from American Type Culture Collection (ATCC, Rockville, MD), cultured in DMEM medium, Dulbecco's Modified Eagle Medium, (Invitrogen Canada Inc. Burlington, ON, Canada) supplemented with 10% FBS, 1ml gentamicin, and incubated in a 5% CO_2_ chamber at 37°C. An immortalized human liver cell line (THLE-3) was purchased from the American Type Culture Collection (ATCC, Rockville, MD). The cells were maintained in precoated flasks with a mixture of bronectin (0.01 mg/mL), bovine collagen type 1 (0.03 mg/mL), and bovine serum albumin (0.01 mg/mL) dissolved in BEGM medium, bronchial tracheal epithelial cell growth medium, and incubated at 37°C and 5% CO2.

### Human mitochondrial v2.0 Oligonucleotide Microarray

The MitoChip v2.0 was obtained from Affymetrix (commercially available GeneChip Human Mitochondrial Resequencing Array 2.0; Santa Clara, CA). Sequences comprising both strands of the entire human mitochondrial genome were synthesized as overlapping 25 bp oligonucleotides probe of defined sequence. To query any given site from human mitochondrial reference sequence, four features (A, C, G and T) are tiled on the MitoChip. The four features differ only by the 13^th^ base, which consists of each of the four possible nucleotides.

### MitoChip Preparation

The total DNA was extracted from cell lines by using the QIAamp DNA Mini kit (Qiagen, Inc., Valencia, CA) according to manufacturer’s instructions. The final DNA was dissolved in bi-distilled water and kept frozen at −20°C until use. Three pairs of overlapping primers used to amplify the entire 16.6-kb mitochondrial genome. The three primer sets were F1: 5'-ATA GGG GTC CCT TGA CCA CCA TCC TCC GT-3' and R1: 5'-GAG CTG TGC CTA GGA CTC CAG CTC ATG CGC CG-3', F2: 5'-CCG ACC GTT GAC TAT TCT CTA CAA ACC AC-3' and R2: 5'- GAT CAG GAG AAC GTG GTT ACT AGC ACA GAG AG- 3', F3: 5'-CAT TCT CAT AAT CGC CCA CGG GCT TAC ATC C-3' and R3: 5'-GTT CGC CTG TAA TAT TGA ACG TAG GTG CC-3'. Briefly, long-range PCR was performed using three PCR primer sets that can amplify the entire mtDNA, using 100 ng of input DNA for each reaction, 5U LA Taq polymerase (TaKara, Mississauga, ON, CA), 5 µL buffer, 2.5 mM each of dNTPs, 0.2 µM of primers were mixed with dH_2_0 to a final reaction volume of 50 µL. The cycling conditions for all reactions were: (1) 95°C for 2 min; (2) 95°C for 15 seconds; (3) 68°C for 7 min; (4) repeat step 2 for 29 times; (5) final extension for 12 min. As a control for PCR amplification and subsequent hybridization, a 7.5 kb plasmid DNA (Tag IQ-EX template) was amplified concomitantly with the test samples, using forward and reverse primers included in the CustomSeq kit (Affymetrix; Santa, CA). The PCR product was purified by using QIAQuick PCR Clean up kit (Qiagen, Inc., Valencia, CA). Spectrophotometric analysis was used to determine the concentration (ng/µL) of the purified PCR product by measuring the absorbance at 260 nm (NanoDrop 1000 Spectrophotometer, Thermo Scientific, Wilmington, USA). The pooled 35 µl of DNA fragments were then digested with DNase I (0.2 U of DNase I/µg DNA) for 15 min in a 50µl reaction (Affymetrix, Cat. 900447). Samples were then incubated at 95°C for 15 min to inactivate DNase I. Fragmented DNA was labeled by adding 2.0 µl of GeneChip DNA labeling reagent and 3.4µl of 30 U/µl terminal deoxynucleotidyl transferase (both from Affymetrix; Santa, CA). The labeling conditions were 37°C 90 min and 95°C 15 min. Prehybridization, hybridization, washing, and scanning of the MitoChip were performed as described in the Affymetrix CustomSeq Resequencing protocol. Hybridization, washing and scanning were performed in array core laboratory at Benaroya Research Institute as described in Affymetrix CustomSeq Resequencing protocol [14].

### Automated Batch Analysis of Microarray Data

Analysis of microarray data for the v2.0 MitoChips was done using GeneChip Sequence Analysis Software (GSEQ) v4.0 (Affymetrix; Santa, CA). GSEQ uses an objective statistical framework, based upon the ABACUS algorithm to assign base calls to each position which meets quality criteria in the mitochondrial genome [14]. GSEQ assigned a base call at any position by using International Union of Pure and Applied Chemistry codes (IUPAC) afterward compared the base calls to the Cambridge reference sequence (rCRS). The base calls assigned either as a homoplasmic variant (mtDNA copies either wild-type or mutant in the same individual), heteroplasmic sequence variant (the mutant and wild-type of mtDNA copies coexist in the same individual), or N-call [15]. Those not recorded in MitoMap (http://www.mitomap.org/MITOMAP) database were categorized as novel mtDNA variations, and those appear in the database were categorized as reported mutation. GSEQ software was used per manufacturer's instructions, with Genome model set to “diploid” and Quality Score threshold set to “3” to maximize base call percentage and fidelity. All variants were annotated using NGS-SNP [16]. For each variant, the program assigns whether it is a coding variant or not (non-synonymous (missense) or synonymous). For non-synonymous variants, SIFT and PolyPhen prediction was obtained. SIFT predicts whether an amino acid substitution affects protein function, which is based on the degree of conservation of amino acid residues in sequence alignments derived from closely related sequences [17]. And PolyPhen predicts possible impact of an amino acid substitution on the structure and function of a human protein using straightforward physical and comparative considerations [18].

### Determination of MtDNA Copy Number

Mitochondrial DNA copy number was measured by a real-time PCR that uses the mitochondrial *ND1* gene as a marker of the entire mtDNA genome and normalized by simultaneous measurement of nuclear DNA encoded *β - actin* gene. DNA was harvested from confluent cultures of HuCCT1, HuH28, OZ, HepG2, Huh-7 and THLE-3 cells using the DNeasy tissue kit followed manufacturer’s instructions (Qiagen, Inc., Valencia, CA). The DNA was quantified by spectrophotometry. The DNA abundance was measured using the TaqMan Universal PCR Master Mix with gene-specific MGB probe labeled with FAM and VIC fluorescent dyes (Applied Biosystems, Burlington, ON, Canada) using the StepOnePlus-Real time PCR System (Applied Biosystems, Burlington, ON, Canada). The sequences of the primers are: *â-actin* L-strand primer: 5'-CATGTGCAAGGCCGGCTTCG-3', *â-actin* H-strand primer: 5'-CTGGGTCATCTTCTCGCGGT-3', *ND1* L-strand primer: 5'-TCTCACCATCGCTCTTCTAC-3', and *ND1* H-strand primer: 5'-TTGGTCTCTGCTAGTGTGGA-3'. Real-time PCR amplification was performed in 20 µl containing 2X TaqMan Universal PCR Master Mix, No AmpErase UNG, 1 µl 20X VIC-RNase P Assay, 1 µl 20X FAM-labeled gene specific assay, 8 µl DNase-free water, 200 nM each primer and 1 µl of each analyzed DNA sample. The concentration of each analyzed DNA sample was 50 ng/µl always. The PCR reactions (20 µL) were carried out in triplicate. The thermal cycling conditions were as follows: 95°C for 20sec followed by 40 cycles of 95°C for 1 sec and 60°C for 20sec. Threshold Cycle (CT) values for each time point were calculated by subtracting the CT of the endogenous gene from the CT of the target gene. Relative quantification values were determined using the 2 ^−ΔΔCT^ methods and expressed as fold change in control (THLE-3) versus other cell lines. The content of *ND1* gene was normalized with the content of *â-actin* gene (nDNA) to calculate the relative mtDNA copy number in each sample.

### Western Blotting

The cultured cells were scraped in PBS and centrifuged at 670×*g* for 5 min. Cell pellets were lysed in 1× RIPA (radio-immuno-precipitation assay) buffer [25 mM Tris·HCl (pH 7.6), 150 mM NaCl, 1% Nonidet P-40, 1% sodium deoxycholate, and 0.1% SDS] supplemented with 1x protease inhibitor. Total protein was quantitated using the BCA (bicinchoninic acid) protein assay (Pierce, Thermo Scientific, ON, Canada). Western blot analysis was performed by using standard techniques. Fifty µg of protein were separated on gradient 4–20% SDS PAGE gels (Bio-Rad, Hercules, CA) and transferred into polyvinylidene fluoride (PVDF) membranes. Membranes then were incubated in tris-buffered saline (TTBS) supplemented with 5% non-fat dry milk for 3 hr at room temperature. Membranes were probed overnight with the OXPHOS cocktail of antibodies (1∶200 dilutions, Mitoscience, Cambridge, Massachusetts, USA) targets the following proteins: 20-kD subunit of Complex I (20 kD), COX II of Complex IV (22 kD), 30-kD subunit of Complex II (30 kD), core 2 of complex III (50 kD), and F1α (ATP synthase) of Complex V (60 kD). Antibodies were then each probed with their corresponding HRP-conjugated secondary antibody (1∶3,000 dilutions, Vector Laboratories, Burlingame, CA) for 1 hr at room temperature. The blots were visualized by using ECL Western blotting detection reagents (Amersham Biosciences, Piscataway, NJ) and developed on Kodak film (Kodak Graphic Communications Company, Burnaby, BC, Canada) according to the manufacturer’s instructions.

### Transmission Electron Microscopy

Briefly, confluent Petri-dishes plates of each CCA and HCC cell lines were washed in PBS and fixed with 2% glutaraldehyde for 1 hr in 4°C. Cells were scraped off from plates and collected in Eppendorf tubes. After centrifugation, cell pellets were further fixed with osmium tetroxide overnight at room temperature. Afterward, following graded dehydration, pellets were epon-embedded and ultra-thin sectioned. Sections were mounted on metal mesh grids, and contrasted with uranyl acetate and lead citrate. Grids were examined in Hitachi H-7650 transmission electron microscope (TEM) (Hitach High-technologies Canada Inc., Rexdale, ON, Canada).

### Measurement of Mitochondrial ΔΨm

The mitochondrial membrane potential sensor JC-1 (5,5', 6,6'-Tetrachloro-1,1',3,3'-tetraethyl-imidacarbocyanine iodide; Molecular Probes, Invitrogen, Germany) was used to label mitochondria in transmembrane manner. The JC-1 stock solution (5 µg/ml) was prepared in anhydrous dimethyl sulphoxide (DSMO) and diluted in supplemented culture medium with final concentration of five µg/ml. In physiologically polarized cells, JC-1 accumulates at mitochondria as red fluorescent J-aggregates while in depolarized cells the dye forms green fluorescent monomers. The labeling procedure was applied following the heat treatments according to the manufacturer's recommendations. A confocal microscopy assay was performed with laser scanning confocal microscopy (Zeiss LSM 510) connected to an inverted microscope with a X40 water- immersion objective lens, optimal laser lines and filter. The fluorescence of TMRE was excited at 564 nm and emitted signals were collected through 600 nm long pass filter. Images were digitized at 8 bits and analyzed using ZEN 2009 software (Carl Zeiss, Toronto, ON, Canada).

### Lactate Measurements

Lactate release was measured using a lactate assay kit from Eton Bioscience, Inc, San Diego, USA (cat no. 1200012002). Briefly, two days before the experiment, cells were seeded in 10 cm^2^ dishes. At the time of measurement, cell density was about 60–70% confluent. Media from the dishes were collected and diluted to 50-fold with double-distilled water. Standard graphs were generated using different concentrations of lactate in 50 µl of volume and 50 µl of lactate assay solution in 96-well plates followed by incubation in a humidified chamber at 37°C for 30 min. Similarly, different dilutions of media (2, 4, 6, 8, and 10 µl/ml) were used to measure extracellular lactate in the linear range. The reaction was stopped by adding 50 µl of 0.5 M acetic acid and absorbance was measured at 490 nm using Synergy HT Microplate Reader.

### NAD^+^/NADH Ratio Assay

Cells were grown to 70% confluence in a 10cm^2^ tissue culture plate in appropriate media (as described above). NADH and NAD^+^ were determined according to manufacturer's protocol (E2ND-100, Bioassay Systems, Hayward, CA, USA). Briefly, cells were counted by trypan blue exclusion method, which determine the number of viable cells present in a cell suspension. Cell pellets were resuspended in 1.5 mL Eppendorf tubes with either 100 µl NAD^+^ extraction buffer (containing 0.40% hydrochloric acid) for NAD^+^ determination or 100 µl NADH extraction buffer (containing 0.40% sodium hydroxide) for NADH determination. Extracts were heated for five min at 60°C and 20 µl of assay buffer (containing 3.0% Tris hydroxymethyl aminomethane and 0.10% BSA) was added followed by the extraction buffer to neutralize the extracts. Mixtures were vortexed and centrifuged at 13,000 rpm for five min. Supernatants were added to working reagent containing 60 µl assay buffer, 1 µl alcohol dehydrogenase, 1 µl ethanol, 14 µl phenazine methosulfate (PMS) and 14 µl tetrazolium dye (MTT). Optical density at 565 nm was recorded at time zero and at 15 min using a 96-well plate reader spectrophotometer (XMark, Bio-Rad, Mississauga, ON, Canada). The difference in absorbance was compared with standard solutions and used to calculate NADH and NAD^+^ concentrations according to manufacturer’s instructions and international standards.

### Statistical Analysis

Data are expressed as mean±SEM, and the number of cells or experiments is shown as n. Statistical comparisons were made with the use of Student ***t*** test. All statistical results were calculated with SPSS 16.0 software and a P value <0.05 was considered as significant. The asterisk is to indicate differences compared to control cell line (THLE-3).

## Results

### Mitochip Analysis

**Figure 1:**
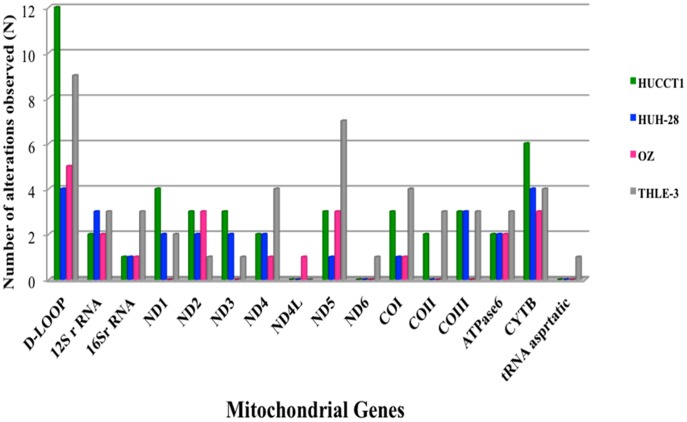
MitoChip Analysis for CCA and Immortalized Hepatocyte Cell Lines. Of the 98 alterations observed in the three cell lines, 24 were non-synonymous coding region alterations (i.e., resulting in an amino acid change), 43 synonymous and 31 involved in non-coding including ribosomal and transfer RNA. Of the 51 alterations observed in Papova-immortalized normal hepatocyte cell line (THLE-3), 10 were non-synonymous coding region alterations, 23 were synonymous and 17 alterations were involved in non-coding, including ribosomal and transfer RNA and one was novel alteration.

To define the pattern and frequency of mtDNA mutations in CCA, the full amount of 226,536 mtDNA bases were sequenced in the three cell lines with a median call rate of 97.90% (Table 1). Consequently, all three cell lines demonstrated at least 24 mtDNA alterations. Of the 102 mtDNA alterations observed in the three CCA cell lines, 38 were synonymous and 30 of them were in non-coding sequences, including ribosomal and transfer RNAs (Table 2). Twenty-eight alterations were non-synonymous coding region mutations, resulting in an amino acid change. Of the 51 alterations observed in Papova-immortalized normal hepatocyte cell lines (THLE-3), 11 were non-synonymous coding region alterations (Table 3), 22 were synonymous variations, 17 alterations were involved in non-coding, including ribosomal and transfer RNAs and one was a novel alteration (Table 2). Among these alterations, three heteroplasmic variations in each of HuCCT1 and in Huh-28 were found to be novel. Remarkably, one of these unique alterations was present in Huh-28. This alteration, T16315G, was located in the control region of mtDNA, which contains silent variations without amino acid changes. The remaining novel alterations were located in protein coding regions, three in the *ND3* gene, one in *ATPase6* and one in *COI*. All of these alterations caused amino acid changes in highly conserved residues (Table 4). Overall, the mtDNA alterations most commonly present in all cell lines, which were moderately invasive and metastasizing lines, involved the *D-loop, 12S rRNA, 16S rRNA, ND2, ND4, COI, ATPase6* and *CYTB* genes (Fig. 1). Additionally, we identified two regions of the mitochondrial genome, the *D-loop* and *CYTB*, which are selectively mutated at a higher rate than other genes. Surprisingly, the metastasizing cell line, OZ, displays no mutation in the genes *ND1, ND3, ND6, CII* and *CIII*, which are mainly present in Complex I and Complex IV. However, OZ is the only cell line that contains a mutation in *ND4L* (Table 2).

**Table 1:** Summary of Array-based Analysis for mtDNA Alterations.

Number of sample analyzed =	6
mtDNA bases per MitoChip v2.0 microarray =	16,569
Total number of mtDNA bases sequenced =	226,536
Total number of mtDNA bases sequenced(assigned by genotyping software) =	163,348
Percent overall bases call rate =	97%
Range of bases called across 6 arrays =	95.6%–99.5%
Median base call rate =	97.90%

**Table 2:** mtDNA alterations in Cholangiocellular Carcinoma (CCA) and Hepatocellular Carcinoma (HCC) Cell Lines.

Cell lines	NTD Pos	Gene location	RCRS sequences	Alteration	Amino acid	Homoplasmy OR Heteroplasmy	Functional_Class
HUCCT1							
	39	D-loop	C	T	NA	Homoplasmy mutation	upstream_gene_variant
	64	D-loop	C	T	NA	Homoplasmy mutation	upstream_gene_variant
	150	D-loop	C	T	NA	Homoplasmy mutation	upstream_gene_variant
	199	D-loop	T	C	NA	Homoplasmy mutation	upstream_gene_variant
	263	D-loop	A	G	NA	Homoplasmy mutation	upstream_gene_variant
	331	D-loop	A	C	NA	Heteroplasmy mutation	upstream_gene_variant
	333	D-loop	T	C	NA	Homoplasmy mutation	upstream_gene_variant
	488	D-loop	A	C	NA	Heteroplasmy mutation	upstream_gene_variant
	489	D-loop	T	C	NA	Homoplasmy mutation	upstream_gene_variant
	750	12S rRNA	A	G	NA	Homoplasmy mutation	non_coding_exon_variant
	1438	12S rRNA	A	G	NA	Homoplasmy mutation	non_coding_exon_variant
	2706	16S rRNA	A	G	NA	Homoplasmy mutation	non_coding_exon_variant
	4071	ND1	C	T	Y-Y	Homoplasmy mutation	synonymous_variant
	4164	ND1	A	G	M-M	Homoplasmy mutation	synonymous_variant
	4769	ND2	A	G	M-M	Homoplasmy mutation	synonymous_variant
	5351	ND2	A	G	L-L	Homoplasmy mutation	synonymous_variant
	6455	COI	C	T	F-F	Homoplasmy mutation	synonymous_variant
	6680	COI	T	C	T-T	Homoplasmy mutation	synonymous_variant
	**7028**	COI	C	T	A-A	Homoplasmy mutation	synonymous_variant
	7684	COII	T	C	L-L	Homoplasmy mutation	synonymous_variant
	9540	COIII	T	C	L-L	Homoplasmy mutation	synonymous_variant
	9824	COIII	T	C	L-L	Homoplasmy mutation	synonymous_variant
	10400	ND3	C	T	T-T	Homoplasmy mutation	synonymous_variant
	10873	ND4	T	C	P-P	Homoplasmy mutation	synonymous_variant
	**11719**	ND4	G	A	G-G	Homoplasmy mutation	synonymous_variant
	12405	ND5	C	T	L-L	Homoplasmy mutation	synonymous_variant
	12705	ND5	C	T	I-I	Homoplasmy mutation	synonymous_variant
	14783	CYB	T	C	L-L	Homoplasmy mutation	synonymous_variant
	15043	CYB	G	A	G-G	Homoplasmy mutation	synonymous_variant
	15301	CYB	G	A	L-L	Homoplasmy mutation	synonymous_variant
	16126	D-loop	T	G	NA	Heteroplasmy mutation	upstream_gene_variant
	16129	D-loop	G	A	NA	Homoplasmy mutation	upstream_gene_variant
	16223	D-loop	C	T	NA	Homoplasmy mutation	upstream_gene_variant
HUH28							
	263	D-loop	A	G	NA	Homoplasmy mutation	upstream_gene_variant
	489	D-loop	T	C	NA	Homoplasmy mutation	upstream_gene_variant
	750	12S rRNA	A	G	NA	Homoplasmy mutation	non_coding_exon_variant
	1041	12S rRNA	A	G	NA	Homoplasmy mutation	non_coding_exon_variant
	1438	12S rRNA	A	G	NA	Homoplasmy mutation	non_coding_exon_variant
	2706	16S rRNA	A	G	NA	Homoplasmy mutation	non_coding_exon_variant
	4769	ND2	A	G	M-M	Homoplasmy mutation	synonymous_variant
	**7028**	COI	C	T	A-A	Homoplasmy mutation	synonymous_variant
	9242	COIII	A	G	K-K	Homoplasmy mutation	synonymous_variant
	9540	COIII	T	C	L-L	Homoplasmy mutation	synonymous_variant
	10400	ND3	C	T	T-T	Homoplasmy mutation	synonymous_variant
	**11719**	ND4	G	A	G-G	Homoplasmy mutation	synonymous_variant
	12705	ND5	C	T	I-I	Homoplasmy mutation	synonymous_variant
	14308	ND6	T	C	G-G	Homoplasmy mutation	synonymous_variant
	14783	CYB	T	C	L-L	Homoplasmy mutation	synonymous_variant
	15301	CYB	G	A	L-L	Homoplasmy mutation	synonymous_variant
	16223	D-loop	C	T	NA	Homoplasmy mutation	upstream_gene_variant
	16316	D-loop	A	G	NA	Homoplasmy mutation	upstream_gene_variant
OZ							
	73	D-loop	A	G	NA	Homoplasmy mutation	upstream_gene_variant
	94	D-loop	G	A	NA	Homoplasmy mutation	upstream_gene_variant
	263	D-loop	A	G	NA	Homoplasmy mutation	upstream_gene_variant
	750	12S rRNA	A	G	NA	Homoplasmy mutation	non_coding_exon_variant
	1438	12S rRNA	A	G	NA	Homoplasmy mutation	non_coding_exon_variant
	2706	16S rRNA	A	G	NA	Homoplasmy mutation	non_coding_exon_variant
	4769	ND2	A	G	M-M	Homoplasmy mutation	synonymous_variant
	5147	ND2	G	A	T-T	Homoplasmy mutation	synonymous_variant
	5417	ND2	G	A	Q-Q	Homoplasmy mutation	synonymous_variant
	**7028**	COI	C	T	A-A	Homoplasmy mutation	synonymous_variant
	8841	ATPase6	C	T	A-A	Homoplasmy mutation	synonymous_variant
	10607	ND4L	C	T	L-L	Homoplasmy mutation	synonymous_variant
	**11719**	ND4	G	A	G-G	Homoplasmy mutation	synonymous_variant
	12501	ND5	G	A	M-M	Homoplasmy mutation	synonymous_variant
	12705	ND5	C	T	I-I	Homoplasmy mutation	synonymous_variant
	14893	CYB	A	G	L-L	Homoplasmy mutation	synonymous_variant
	16223	D-loop	C	T	NA	Homoplasmy mutation	upstream_gene_variant
	16519	D-loop	T	C	NA	Homoplasmy mutation	upstream_gene_variant
HUH7							
	73	D-loop	A	G	NA	Homoplasmy mutation	upstream_gene_variant
	150	D-loop	C	T	NA	Homoplasmy mutation	upstream_gene_variant
	197	D-loop	A	M	NA	Heteroplasmy mutation	upstream_gene_variant
	199	D-loop	T	C	NA	Homoplasmy mutation	upstream_gene_variant
	263	D-loop	A	G	NA	Homoplasmy mutation	upstream_gene_variant
	750	12S rRNA	A	G	NA	Homoplasmy mutation	non_coding_exon_variant
	1438	12S rRNA	A	G	NA	Homoplasmy mutation	non_coding_exon_variant
	2706	16S rRNA	A	G	NA	Homoplasmy mutation	non_coding_exon_variant
	4071	ND1	C	T	Y-Y	Homoplasmy mutation	synonymous_variant
	4164	ND1	A	G	M-M	Homoplasmy mutation	synonymous_variant
	4769	ND2	A	G	M-M	Homoplasmy mutation	synonymous_variant
	5351	ND2	A	G	L-L	Homoplasmy mutation	synonymous_variant
	6455	COI	C	T	F-F	Homoplasmy mutation	synonymous_variant
	6680	COI	T	C	T-T	Homoplasmy mutation	synonymous_variant
	**7028**	COI	C	T	A-A	Homoplasmy mutation	synonymous_variant
	7684	COII	T	C	L-L	Homoplasmy mutation	synonymous_variant
	9540	COIII	T	C	L-L	Homoplasmy mutation	synonymous_variant
	10400	ND3	C	T	T-T	Homoplasmy mutation	synonymous_variant
	**11719**	ND4	G	A	G-G	Homoplasmy mutation	synonymous_variant
	12405	ND5	C	T	L-L	Homoplasmy mutation	synonymous_variant
	12705	ND5	C	T	I-I	Homoplasmy mutation	synonymous_variant
	14783	CYB	T	C	L-L	Homoplasmy mutation	synonymous_variant
	15043	CYB	G	A	G-G	Homoplasmy mutation	synonymous_variant
	15301	CYB	G	A	L-L	Homoplasmy mutation	synonymous_variant
	16129	D-loop	G	A	NA	Homoplasmy mutation	upstream_gene_variant
	16223	D-loop	C	T	NA	Homoplasmy mutation	upstream_gene_variant
HEPG2							
	73	D-loop	A	G	NA	Homoplasmy mutation	upstream_gene_variant
	146	D-loop	T	C	NA	Homoplasmy mutation	upstream_gene_variant
	263	D-loop	A	G	NA	Homoplasmy mutation	upstream_gene_variant
	499	D-loop	G	A	NA	Homoplasmy mutation	upstream_gene_variant
	750	12S rRNA	A	G	NA	Homoplasmy mutation	non_coding_exon_variant
	827	12S rRNA	A	G	NA	Homoplasmy mutation	non_coding_exon_variant
	1438	12S rRNA	A	G	NA	Homoplasmy mutation	non_coding_exon_variant
	2706	16S rRNA	A	G	NA	Homoplasmy mutation	non_coding_exon_variant
	4755	ND2	T	C	L-L	Homoplasmy mutation	synonymous_variant
	4769	ND2	A	G	M-M	Homoplasmy mutation	synonymous_variant
	4820	ND2	G	A	E-E	Homoplasmy mutation	synonymous_variant
	4976	ND2	A	C	G-G	Heteroplasmy mutation	synonymous_variant
	4977	ND2	T	C	L-L	Homoplasmy mutation	synonymous_variant
	6473	COI	C	T	I-I	Homoplasmy mutation	synonymous_variant
	**7028**	COI	C	T	A-A	Homoplasmy mutation	synonymous_variant
	7241	COI	A	G	A-A	Homoplasmy mutation	synonymous_variant
	9950	COIII	T	C	V-V	Homoplasmy mutation	synonymous_variant
	**11719**	ND4	G	A	G-G	Homoplasmy mutation	synonymous_variant
	13590	ND5	G	A	L-L	Homoplasmy mutation	synonymous_variant
	16217	D-loop	T	C	NA	Homoplasmy mutation	upstream_gene_variant
	16519	D-loop	T	C	NA	Homoplasmy mutation	upstream_gene_variant
THLE-3							
	73	D-loop	A	G	NA	Homoplasmy mutation	upstream_gene_variant
	146	D-loop	T	C	NA	Homoplasmy mutation	upstream_gene_variant
	152	D-loop	T	C	NA	Homoplasmy mutation	upstream_gene_variant
	193	D-loop	A	C	NA	Heteroplasmy mutation	upstream_gene_variant
	195	D-loop	T	C	NA	Homoplasmy mutation	upstream_gene_variant
	263	D-loop	A	G	NA	Homoplasmy mutation	upstream_gene_variant
	750	12S rRNA	A	G	NA	Homoplasmy mutation	non_coding_exon_variant
	1018	12S rRNA	G	A	NA	Homoplasmy mutation	non_coding_exon_variant
	1438	12S rRNA	A	G	NA	Homoplasmy mutation	non_coding_exon_variant
	2416	16S rRNA	T	C	NA	Homoplasmy mutation	non_coding_exon_variant
	2706	16S rRNA	A	G	NA	Homoplasmy mutation	non_coding_exon_variant
	2789	16S rRNA	C	T	NA	Homoplasmy mutation	non_coding_exon_variant
	3594	ND1	C	T	V-V	Homoplasmy mutation	synonymous_variant
	4104	ND1	A	G	L-L	Homoplasmy mutation	synonymous_variant
	4769	ND2	A	G	M-M	Homoplasmy mutation	synonymous_variant
	5581	NC4	A	G	NA	Homoplasmy mutation	upstream_gene_variant
	**7028**	COI	C	T	A-A	Homoplasmy mutation	synonymous_variant
	7175	COI	T	C	T-T	Homoplasmy mutation	synonymous_variant
	7256	COI	C	T	N-N	Homoplasmy mutation	synonymous_variant
	7274	COI	C	T	G-G	Homoplasmy mutation	synonymous_variant
	7521	tRNA aspartic acid	G	A	NA	Homoplasmy mutation	non_coding_exon_variant
	7771	COII	A	G	E-E	Homoplasmy mutation	synonymous_variant
	9221	COIII	A	G	S-S	Homoplasmy mutation	synonymous_variant
	9540	COIII	T	C	L-L	Homoplasmy mutation	synonymous_variant
	10115	ND3	T	C	I-I	Homoplasmy mutation	synonymous_variant
	**11719**	ND4	G	A	G-G	Homoplasmy mutation	synonymous_variant
	11914	ND4	G	A	T-T	Homoplasmy mutation	synonymous_variant
	11944	ND4	T	C	L-L	Homoplasmy mutation	synonymous_variant
	12693	ND5	A	G	K-K	Homoplasmy mutation	synonymous_variant
	12705	ND5	C	T	I-I	Homoplasmy mutation	synonymous_variant
	13590	ND5	G	A	L-L	Homoplasmy mutation	synonymous_variant
	13650	ND5	C	T	P-P	Homoplasmy mutation	synonymous_variant
	13803	ND5	A	G	T-T	Homoplasmy mutation	synonymous_variant
	14566	ND6	A	G	G-G	Homoplasmy mutation	synonymous_variant
	15301	CYB	G	A	L-L	Homoplasmy mutation	synonymous_variant
	15784	CYB	T	C	P-P	Homoplasmy mutation	synonymous_variant
	16223	D-loop	C	T	NA	Homoplasmy mutation	upstream_gene_variant
	16309	D-loop	A	G	NA	Homoplasmy mutation	upstream_gene_variant
	16390	D-loop	G	A	NA	Homoplasmy mutation	upstream_gene_variant

Note: RCRS, Revised Cambridge Reference Sequence; both RCRS position and reference nucleotide at that specific position are designed as above. In particular, the highly specific MitoAnalyzer tool (http://www.cstl.nist.gov/biotech/strbase/mitoanalyzer-direction.html) was used for determining the effect of base substitution on translated protein sequence. The bold base positions are recurrent alterations present in all CCA and HCC cell lines and hepatocyte cell line.

**Table 3:** Non-Synonymous Mitochondrial DNA and Amino Acid Alterations.

Cell lines	NTD Pos	Gene location	RCRS sequences	Alteration	Amino acid	Homoplasmy OR Heteroplasmy	Functional_Class
HUCCT1							
	3308	ND1	T	C	M-T	Homoplasmy mutation	initiator_codon_variant
	4048	ND1	G	A	D-T	Homoplasmy mutation	missense_variant
	5460	ND2	G	A	A-T	Homoplasmy mutation	missense_variant
	7853	COII	G	A	V-I	Homoplasmy mutation	missense_variant
	8701	ATPase6	A	G	M-V	Homoplasmy mutation	missense_variant
	**8860**	ATPase6	A	G	T-A	Homoplasmy mutation	missense_variant
	9539	COIII	A	C	Q-H	Homoplasmy mutation	missense_variant
	10345	ND3	T	C	I-T	Homoplasmy mutation	missense_variant
	10398	ND3	A	G	T-A	Homoplasmy mutation	missense_variant
	12811	ND5	T	C	Y-H	Homoplasmy mutation	missense_variant
	**14766**	CYB	C	T	T-I	Homoplasmy mutation	missense_variant
	**15326**	CYB	A	G	T-A	Homoplasmy mutation	missense_variant
	15756	CYB	G	A	W-TER	Heteroplasmy mutation	stop_gained
HUH28							
	3394	ND1	T	C	Y-M	Homoplasmy mutation	missense_variant
	3407	ND1	G	A	R-H	Heteroplasmy mutation	missense_variant
	4491	ND2	G	A	L-I	Homoplasmy mutation	missense_variant
	8701	ATPase6	A	G	T-A	Homoplasmy mutation	missense_variant
	**8860**	ATPase6	A	G	T-A	Homoplasmy mutation	missense_variant
	9243	COIII	C	G	P-A	Heteroplasmy mutation	missense_variant
	10398	ND3	A	G	T-A	Homoplasmy mutation	missense_variant
	11963	ND4	G	A	V-I	Homoplasmy mutation	missense_variant
	**14766**	CYB	C	T	T-I	Homoplasmy mutation	missense_variant
	**15326**	CYB	A	G	T-A	Homoplasmy mutation	missense_variant
							
OZ	**8860**	ATPase6	A	G	T-A	Homoplasmy mutation	missense_variant
	11016	ND4	G	A	S-N	Homoplasmy mutation	missense_variant
	13183	ND5	A	G	I-V	Homoplasmy mutation	missense_variant
	**14766**	CYB	C	T	T-I	Homoplasmy mutation	missense_variant
	**15326**	CYB	A	G	T-A	Homoplasmy mutation	missense_variant
HUH7							
	3392	ND1	G	A	G-D	Heteroplasmy mutation	missense_variant
	4048	ND1	G	A	D-G	Homoplasmy mutation	missense_variant
	5460	ND2	G	A	A-T	Homoplasmy mutation	missense_variant
	8701	ATPase6	A	G	M-V	Homoplasmy mutation	missense_variant
	**8860**	ATPase6	A	G	T-A	Homoplasmy mutation	missense_variant
	9539	COIII	A	C	Q-H	Heteroplasmy mutation	missense_variant
	10345	ND3	T	C	I-T	Homoplasmy mutation	missense_variant
	10398	ND3	A	G	T-A	Homoplasmy mutation	missense_variant
	12811	ND5	T	C	Y-H	Homoplasmy mutation	missense_variant
	13276	ND5	A	M	M-L	Heteroplasmy mutation	missense_variant
	**14766**	CYB	C	T	T-I	Homoplasmy mutation	missense_variant
	**15326**	CYB	A	G	T-A	Homoplasmy mutation	missense_variant
	15758	CYB	A	G	I-V	Homoplasmy mutation	missense_variant
HEPG2							
	3547	ND1	A	G	I-V	Homoplasmy mutation	missense_variant
	**8860**	ATPase6	A	G	T-A	Homoplasmy mutation	missense_variant
	11177	ND4	C	T	P-S	Homoplasmy mutation	missense_variant
	14757	CYB	T	C	M-T	Homoplasmy mutation	missense_variant
	**14766**	CYB	C	T	T-I	Homoplasmy mutation	missense_variant
	**15326**	CYB	A	G	T-A	Homoplasmy mutation	missense_variant
THLE-3							
	7173	COI	A	C	T-A	Heteroplasmy mutation	missense_variant
	7770	COII	A	G	E-G	Heteroplasmy mutation	missense_variant
	8701	ATPase6	A	G	T-A	Homoplasmy mutation	missense_variant
	**8860**	ATPase6	A	G	T-A	Homoplasmy mutation	missense_variant
	9539	COIII	A	C	Q-H	Heteroplasmy mutation	missense_variant
	10398	ND3	A	G	T-A	Homoplasmy mutation	missense_variant
	11945	ND4	A	C	T-P	Heteroplasmy mutation	missense_variant
	12694	ND5	T	G	Y-D	Heteroplasmy mutation	missense_variant
	13805	ND5	C	G	A-G	Heteroplasmy mutation	missense_variant
	**14766**	CYB	C	T	T-I	Homoplasmy mutation	missense_variant
	**15326**	CYB	A	G	T-A	Homoplasmy mutation	missense_variant

Notes: The nucleotide numbering system is based on the human mitochondrial revised Cambridge Reference Sequence. The tool on http://sift.jcvi.org was used to predict, whether an amino acid substitution affects protein function. The tool on http://genetics.bwh.harvard.edu/pph/ was used to predict possible impact of an amino acid substitution on the structure and function of a human protein. The bold base positions are recurrent alterations present in all CCA and HCC cell lines and hepatocyte cell line.

**Table 4:** Novel mtDNA Alterations in Cholangiocellular Carcinoma (CCA) and Hepatocellular Carcinoma (HCC) Cell Lines.

Cell lines	NTD Pos	Gene location	RCRS sequences	Alteration	Amino acid	Homoplasmy OR heteroplasmy	NON-synonymous	Functional_Class
HUCCT1								
	6678	COI	A	C	T-P	Heteroplasmy mutation	YES	missense_variant
	9179	ATPase6	T	C	V-A	Heteroplasmy mutation	YES	missense_variant
	10399	ND3	C	G	T-S	Heteroplasmy mutation	YES	missense_variant
HUH28								
	10399	ND3	C	G	T-S	Heteroplasmy mutation	YES	missense_variant
	10401	ND3	G	T	E-TER	Heteroplasmy mutation	YES	stop_gained
	16315	D-loop	T	G	NA	Heteroplasmy mutation	NO	upstream_gene_variant
HUH7								
	1285	12S rRNA	G	T	NA	Heteroplasmy mutation	NO	non_coding_exon_variant
	6678	COI	A	C	T-P	Heteroplasmy mutation	YES	missense_variant
	9179	ATPase6	T	C	V-A	Heteroplasmy mutation	YES	missense_variant
	10399	ND3	C	G	T-S	Heteroplasmy mutation	YES	missense_variant
	13277	ND5	T	C	M-T	Homoplasmy mutation	YES	missense_variant
	15002	CYB	G	A	G-S	New/Homoplasmy mutation	YES	missense_variant
HEPG2								
	4759	ND2	T	C	M-T	Heteroplasmy mutation	YES	missense_variant
	14756	CYB	A	C	M-L	Heteroplasmy mutation	YES	missense_variant
	14772	CYB	C	T	P-L	Heteroplasmy mutation	YES	missense_variant
	16521	D-loop	A	C	NA	Heteroplasmy mutation	NO	upstream_gene_variant
THLE-3								
	2620	16S rRNA	G	A	NA	Heteroplasmy mutation	NO	non_coding_exon_variant

Notes: The nucleotide numbering system is based on the human mitochondrial revised Cambridge Reference Sequence. The tool on http://sift.jcvi.org was used to predict, whether an amino acid substitution affects protein function. The tool on http://genetics.bwh.harvard.edu/pph/ was used to predict possible impact of an amino acid substitution on the structure and function of a human protein.

**Figure 2:**
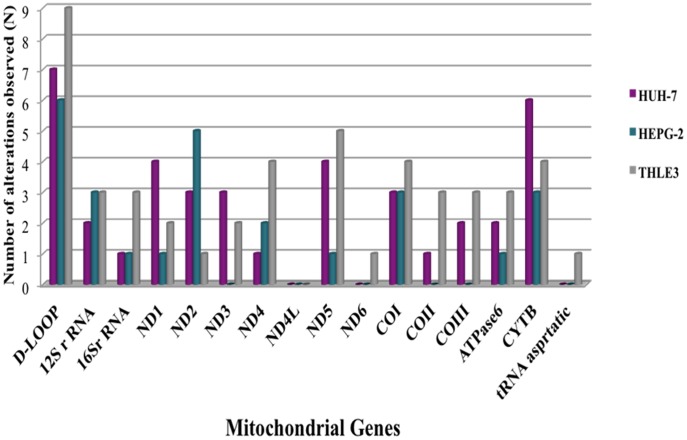
MitoChip Analysis for HCC and Immortalized Hepatocyte Cell Lines. Of the 66 mutations observed in the two cell lines (HEPG-2, HuH-7), 18 were non-synonymous coding region mutations (i.e., resulting in an amino acid change), 28 synonymous and 20 involved in non-coding including ribosomal and transfer RNA. Of the 51 alterations observed in Papova-immortalized normal hepatocyte cell line (THLE-3), 10 were non-synonymous coding region alterations, 23 were synonymous and 17 alterations were involved in non-coding, including ribosomal and transfer RNA and one was novel alteration.

Among the 76 mtDNA alterations observed in the two HCC cell lines, HepG-2 and HuH-7, 19 alterations were non-synonymous coding region mutations resulting in an amino acid change. Additionally, 27 alterations were synonymous, 17 variations were involved in non-coding, including ribosomal, transfer RNA and D-loop (Table 2) and 10 were novel mutations (Table 4). The most common mutations present in both cell lines were involved in the following genes: *D-loop, 12S rRNA, 16S rRNA, ND1, ND2, ND4, ND5, COI, COIII, ATPase6* and *CYTB*. Among these variations, ten alterations were identified as novel in HCC cell lines, six in Huh-7 and four in HepG-2. All these unique alterations were heteroplasmic with the exception of two homoplasmic modifications, which were present in Huh-7’s G15002A *CYB* and T13277C *ND5* genes. The novel heteroplasmic alterations were found in protein coding regions; however, two alterations were present in the *12SRNA* gene in HuH-7 and the control region, or *D-loop*, in HepG-2 (Table 4). Nevertheless, some alterations were only present in HuH-7 cell lines, including *ND3* and *COII* (Fig. 2), while the *ND6* mutation was only present in HuH-28 and absent in both HCC cell lines.

Several recurrent sequence alterations were observed in all CAA, HCC and immortalized hepatocyte cell lines (Table 2). Notably, all of these alterations were homoplasmic and present in *ND4, ND5, COI, CYB* and *ATPase6*. There are three recurrent non-synonymous variants, A8860G, C14766T and A15326G. Not surprisingly, they are not deleterious according to SIFT and PolyPhen prediction. However, the significance of these recurrent alterations in the carcinogenesis and the progression of CCA and HCC is intriguing and worthy to be investigated further.

Besides the recurrent variants, there are also some interesting cell line specific known non-synonymous variants. For example, T3308C in HUCCT1 changes one base of the first codon of ND1. Additionally, both SIFT and PolyPhen predicts the amino acid change from methionine to threonine affects the protein function (probably damaging). G3407A in HUH28, is also predicted to be deleterious by both SIFT and PolyPhen. Another case, G15756A in HUCCT1, adds a stop codon to CYB, which causes 12 amino acids to be missing.

### Alterations in mtDNA Copy Number of CCA and HCC Cell Lines

**Figure 3:**
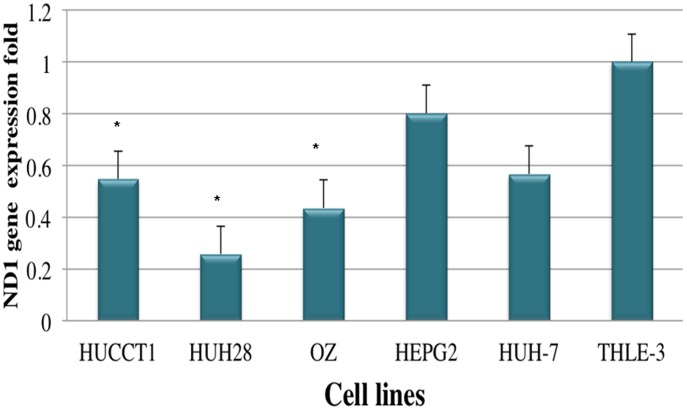
Mitochondrial DNA Copy Number for CAA, HCC and Immortalized Hepatocyte Cell Lines. Relative quantification values were determined by using the 2 −ΔΔCT method and expressed as fold change in control (THLE-3) versus other cell lines. Genes analyzed were β-actin (endogenous gene) and ND1 (gene of interest). All experiments have been performed in triplicate.

To investigate mtDNA copy number changes occurred in CCA and HCC, we further analyzed mtDNA copy number in CCA and HCC cell lines compared with immortalized hepatocyte cell line by real-time PCR. All CCA (HuCCT1, HuH28, OZ) cell lines showed a marked reduction of mtDNA copy number compared with immortalized hepatocyte cell line. The average of mtDNA copy number/β- actin level in CCA was significantly lower than that in immortalized hepatocyte cell line (P<0.05) (P =  .021, P =  .017, P =  .070), respectively (Fig. 3). The hepatocellular carcinoma cell lines (HCC) (HepG-2 and Huh-7) had less total mitochondrial DNA than immortalized hepatocyte cell line; however, the differences between HCC and immortalized hepatocyte cell line were not significant (P =  .437, P =  .083), respectively (Fig. 3).

### Analysis of OXPHOS Proteins

**Figure 4:**
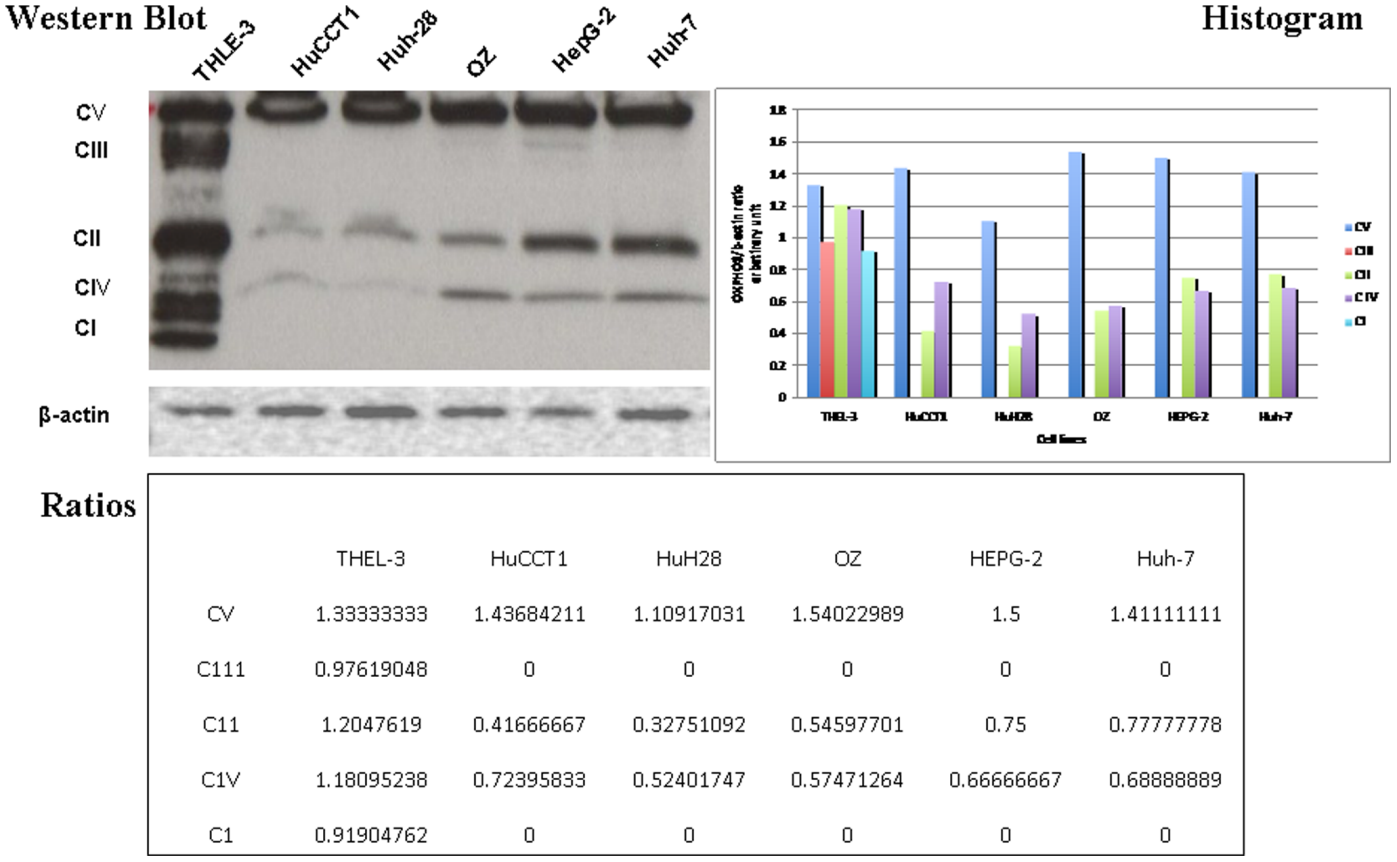
Analysis of OXPHOS Proteins (Western blot, Ratios, and Histograms) for CAA (HuCCT1, HuH-28, OZ), HCC (Hep-G2, Huh-7), and Papova-immortalized Cell Lines of Hepatocytes (THLE-3). Mitochondrial respiratory proteins in CCA and HCC show decreased levels of the nuclear and mitochondrial encoded proteins, complex I and complex III. The housekeeping gene (protein control) is beta-actin. Western blot with ratio and histograms are provided. All experiments have been performed in triplicate.

Respiratory protein content, OXPHOS, was reduced in CCA and HCC. Moreover, immunoblot analyses indicated that the cellular content of the nuclear and mitochondrial encoded proteins, Complex I and Complex III, were reduced in CCA and HCC cell lines when compared with tumor-free hepatocytes (Fig. 4).

### Morphology of Mitochondria in CCA and HCC Cell Lines

**Figure 5:**
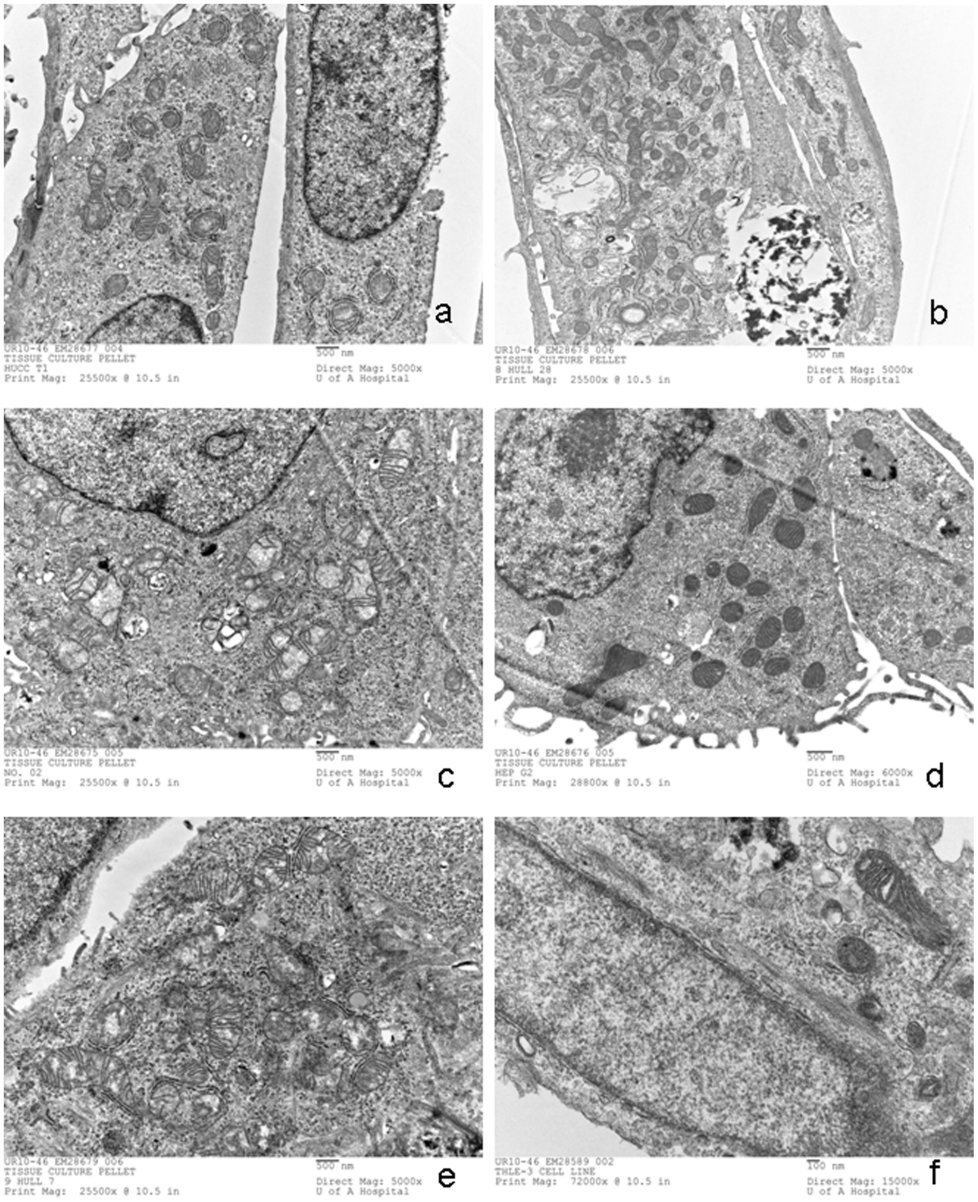
Ultrastructure of the Mitochondria with Alterations of Cristae in HCC, CCA (a-e) and Immortalized Hepatocytes (f). Electron microscopic images for CAA HuCCT1 (a), HuH28 (b), OZ(c), HCC (HepG2 (d), Huh-7 (e) and normal hepatocytes (THLE-3) (f) cell lines.

As shown in HuCCT1 (Fig. 5a) showed primitive mitochondria elements with an oval shape, some of which had very few cristae. HuH-28 (Fig. 5b) exhibited a dense matrix with decreased cristae. OZ (Fig. 5c) cell line has a normal matrix, with very few cristae in both ovular and tubular mitochondria; in addition, dense bodies were found in some mitochondria as well as in cytosol. Interestingly, glycogen material or endoplasmic reticulum surrounded the mitochondria. Also, HepG2 (Fig. 5d) displayed ovular mitochondria with a dense matrix and normal parallel cristae. Finally, HuH-7 (Fig. 5e) showed both tubular and ovular mitochondria, some of which had a decreased number of cristae without dense bodies. All CCA and HCC cell lines were compared with normal hepatocyte cell lines, THLE-3 (Fig. 5f), which showed mostly cristae in tubular mitochondria.

### ΔΨ_m_ of THLE-3 versus CCA and HCC Cell Lines

**Figure 6:**
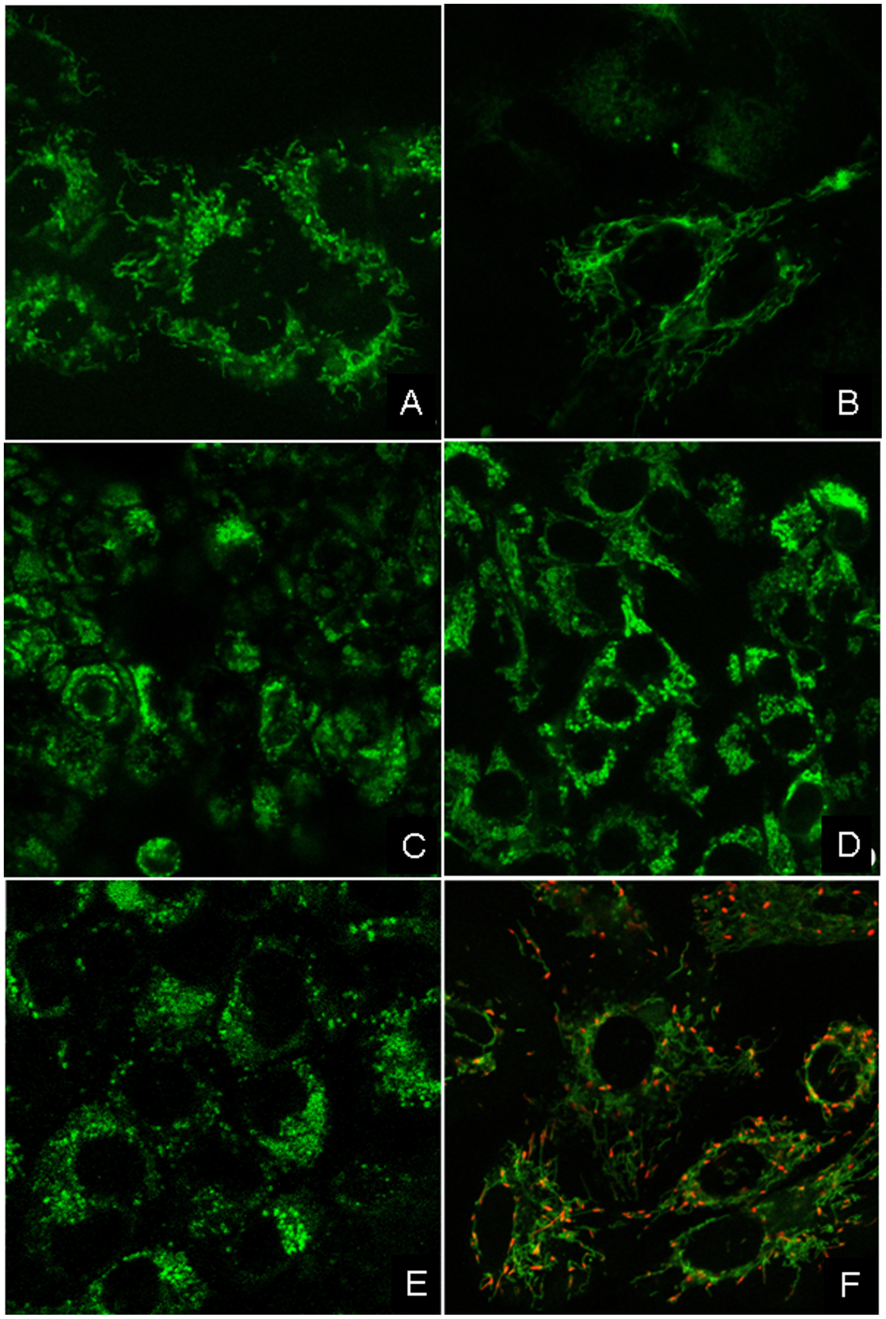
Confocal Images of Mitochondrial Inner Membrane Potential in HCC, CCA (a–e) and Immortalized Cell Lines of Hepatocytes (f). Confocal images of mitochondrial inner membrane potential (ΔΨ_m_) CAA (HuCCT1 (a), HuH28 (b), OZ (c), HCC (HepG2) (d), Huh-7 (e), and normal hepatocyte THLE-3 (f) cell lines. Regions of high mitochondrial polarization are indicated by red fluorescence due to J-aggregate formation by the concentrated dye. Depolarized regions are indicated by the green fluorescence of the JC-1 monomers.

The intensity of the fluorescent TMRE (tetramethylrhodamine ethyl ester) probe was used as an indirect measure of mitochondrial inner membrane potential (ΔΨ_m_) in CCA and HCC cell lines. Immunoflourescence images shown that the normal cell line (Fig. 6f) was hyperpolarized compared to all CAA and HCC cell lines (Fig. 6a–e), which were depolarized of ΔΨ_m_. These results suggested that there were some changes of mitochondrial function in the cancer cell lines.

### Determination of Intracellular Metabolite Concentrations

**Figure 7:**
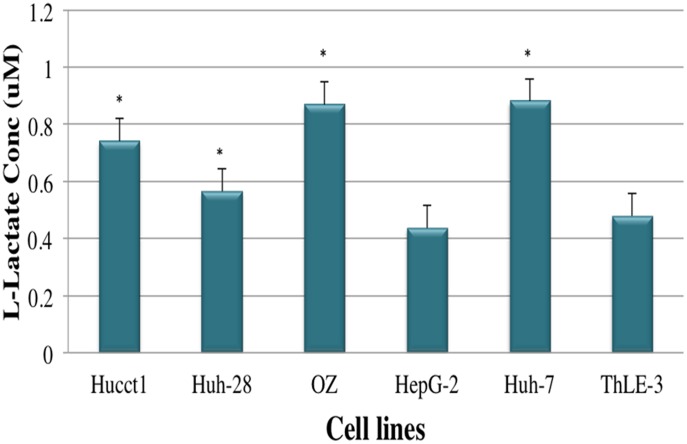
Extracellular Lactate Level for CAA, HCC and Immortalized Cell Lines of Hepatocytes. Extracellular lactate level was determined in the media from 1×10^6^ cells and calculated after 48 h of culturing. Lactate measurements significantly increased in all CCA cells lines and in HuH-7 of HCC cell lines. Data are mean±SEM. Data were confirmed by three independent experiments. *P<0.05 was regarded as significant.

We measured lactate and NAD^+^/NADH ratio in both CCA and HCC cell lines. As lactate is the product of anaerobic glycolysis, mitochondrial dysfunction is usually associated with increased levels of lactate in normal aerobic conditions [19]. As shown in Figure 7, lactate measurements significantly increased in all CCA cells lines and in HuH-7 of HCC cell lines, indicating an increasing adaptation on glycolysis with increasing alterations mtDNA. In addition, All CCA and HCC cell lines except HuH-28 significantly showed high NADH levels compared to normal hepatocytes. The high NADH levels are caused by a disruption of the glycerol 3-phosphate shuttle and a reduction of glutaminolysis as found in breast cancer cell lines (MCF-7 cells) [4].

## Discussion

This study represents the first alternative scanning of the entire mitochondrial genome in cholangiocellular carcinoma (CCA) and hepatocellular carcinoma (HCC) lines to the best of our knowledge. Specifically, we screened the entire mitochondrial genome of three CCA and two HCC cell lines for mitochondrial alterations not only on genetic basis, but also on their morphologic and functional (biochemical) effects. In this process, we used the MitoChip array, comparing it with the revised Cambridge Reference Sequence (rCRS) to evaluate the nucleotide alterations. Because the corresponding non-cancerous cells, which would differentiate mutations from rare polymorphisms, were not available, we relied on MitoChip and rCRS.

Although alterations occurred throughout the entire mitochondrial genome, most of these changes emerged in the D-loop. The mitochondrial D-loop, approximately 1 kb in size, contains *cis*-acting elements involved in regulating the transcription and replication of the mitochondrial genome [20]. We found that all cell lines with mtDNA alterations contained at least one of the D-loop, tRNA, rRNA or non-synonymous coding alterations (Fig. 1,2), hence acquiring the potential for altering mitochondrial function via alterations in transcription, translation and replication, a number of processes tightly linked to carcinogenesis. Our findings of asymmetric alterations in the D-loop are consistent with previous studies showing modifications in the regulation of the mitochondrial genome, which may be associated with carcinogenesis in CCA and HCC [21]. However, our results are unique not only for the cancer type, but also for the morphologic and functional data acquired during this study. Morphologic and biochemical data are supporting molecular data making a solid base for future studies. A related study by Yin P et al 2004 demonstrated a decreased copy number of mtDNA near the D-loop in HCC [21]. In combination, these findings emphasize that alterations in the D-loop region represent indeed important events during the early phase of liver carcinogenesis. Zhang et al illustrated the utility of single nucleotide polymorphisms (SNPs) and mutations in the mitochondria D-loop region for predicting the risk of HCC and for differentiating among HCCs with distinct etiologies [22].

In the coding region, we found 28 CCA and 19 HCC non-synonymous predicted amino acids changes. On the other hand, disproportionate distributions of synonymous alterations were observed throughout the coding regions, involving all complexes (Table 2). The alteration in the *ND4L* gene of the metastasizing cell line, OZ, has also been identified in regions of dysplasia adjacent to primary head and neck tumors, suggesting that mitochondrial alteration occurred early in CCA tumors, prior to the appearance of the invasive phenotype [10]. In cancer, synonymous mtDNA changes affect *in vivo* protein structures and alter functionality that support our data using SIFT and Polyphen tools, which showed defects in protein structure [23], [24]. We found 11 non-synonymous alterations in Papova-immortalized hepatocyte cell line (THLE-3). However, it remains unclear whether these changes occurred because of probably true biological heteroplasmy in liver cells, or more unlikely, were a molecular induction of the Papova-virus immortalization step and subsequent cell passaging.

Despite the presence of certain recurrent alterations, we were unable to hot spot mutations corresponding to studies in pancreas cancer, such as *KRAS2* and *BRAF* [25], [26]. We compared the non-synonymous alterations that we had identified to the mitochondrial genome database [27]. Consequently, several of our reported alterations may exhibit a functional correlation with human diseases. Specifically, we found that G3407A has been associated with an uncommon variety of hypertrophic cardiomyopathy [28]. In addition, we detected an alteration in T12811C that might fulfill a potential modifier role in increasing the penetrance and expressivity of the primary Leber's hereditary optic neuropathy (LHON), a mitochondrial inherited degeneration of retinal ganglion cells and their axons that leads to an acute or subacute loss of central vision with blurring and clouding of vision as first symptoms [29]. In general, this study detected several non-synonymous alterations in specific locations that have been reported in other cancers. We further located A8701G, which was found in thyroid tumors [30]. A10398G had been associated with invasive breast cancer and esophageal cancer in Afro-American individuals [31], [32]. An identical alteration, G15756A, has been reported in human colonic crypt stem cells; this variation is indeed important for understanding of the findings of mtDNA mutations in aging tissues and tumors as well as for determining the frequency of mtDNA mutations within a cell [33]. Our novel non-synonymous alterations, A6678G, T9179Y, C10399S, G10401, T13277C, G15002A, T4759Y, A14756M, and C14772Y might fulfill a function as cancer predisposition. For instance, the A6678G modification changes a moderately conserved threonine residue to proline in *COI*. The alteration of T9179Y changes a highly-conserved hydrophobic valine to a hydrophilic alanine in *ATPase6*. Collectively, these non-synonymous alterations are potentially harmful because they occur in a highly conserved amino acid; therefore, they may affect the mitochondrial OXPHOS function. The common 4977-bp deletion of the mitochondrial genome was not detected by the current array, MitoChip. Consequently, a more comprehensive analysis is required for examining this common deletion in CCA and HCC, since the variation may affect the mutational rate of both normal and tumor tissues [21]. Furthermore, Yin et al 2004 revealed a low frequency of mtDNA 4977-bp deletion in HCC as compared to the corresponding normal tissue. Such findings likely confirmed the contribution of the 4977 deletion in accumulating the two types of mtDNA mutations in HCC [21]. The low abundance of mtDNA has been reported in cancer tissues such as renal [34], gastric [35], breast [36] and hepatocellular carcinoma [21]. Conversely, an increase in mtDNA content was shown in renal oncocytomas [37], head and neck cancer [38], endometrial [39], ovarian [40], and colo-rectal carcinoma [41]. Interestingly, the total mtDNA copy numbers varied between HCC and paired non-neoplastic areas. In particular, these differences depend on the background of the liver. In fact, HCC has fewer mtDNA in non-cirrhotic livers than it does in cirrhotic livers [21], [42]. The alteration of mtDNA content is specific to the type of cancer [30]. Although the effects of these alterations on the carcinogenesis or the tumor progression of CCA and HCC is still obscure, these modifications have the potential to impair the function of the OXPHOS system in CCA and HCC. The underlying molecular mechanisms of the alterations in cancer cell mtDNA are, however, unclear.

Another important objective of the present study was to determine if the morphological alterations in the mitochondria could be correlated with perturbations of mitochondrial function. Accordingly, we measured the enzyme activities of complexes I, II, II + III and IV from CCA and HCC cell lines. In particular, these cell lines demonstrated a marked reduction in complexes I and III (Fig. 4). These complexes play a crucial role in generating an electrochemical potential when the electrons are transferred from ubiquinol to cytochrome *c* in order to synthesize ATP. They are the major sites for ROS production in the electron transport chain. The concomitant reduction in the content of mitochondrial respiratory proteins strongly suggests that the biogenesis of mitochondria is repressed in CCA and HCC [7], [17]. Our findings are consistent with the previous study by Ohashi et al., 2004 [43].

**Figure 8:**
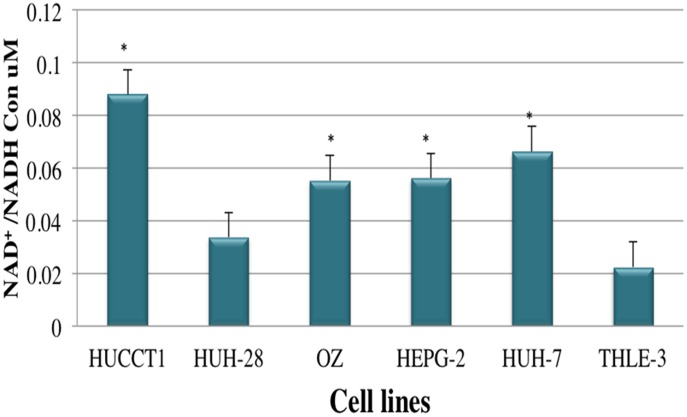
Cytosolic-free NAD^+^/NADH Ratios in CCA and HCC Cell Lines. All CCA and HCC cell lines except HuH-28 significantly showed high NADH levels comparing to normal hepatocyte. Data are mean±SEM. Data were confirmed by three independent experiments. *P<0.05 was regarded as significant.

Moreover, it is important to highlight that there is a direct association between bioenergetics and mitochondrial fusion machinery [44], [45]. Previous studies have demonstrated that the involvement of Mitofusion and/or Opa1 results in severe cellular settings, including cell growth, mitochondrial membrane potential, and cellular respiration. Enhanced respiration seems to correlate with interconnected network and enlarged cristae, while low OXPHOS activity and high glycolysis seems to correlate with smaller mitochondria and reduced intercristal space [44], [45]. Collectively, our findings draw also attention to the substantial changes of mitochondrial shape and its potential effect on tumorigenesis and ultrastructure was key in our investigation as well. The upregulation of ubiquinol-cytochrome *c* reductase suggests the amplification of the ubiquinol-cytochrome *c* reductase gene as found in a variety of tumors along with the elevation of mitochondrial membrane potential (ΔΨ_m_)**.** In 1956, Warburg showed that cancer cells have a respiratory deficiency that is associated with decreased numbers and reduced levels of OXPHOS proteins. Hence, our data seem to support Warburg’s study suggesting that mitochondrial biogenesis may be impaired during liver carcinogenesis [46]. Most cancer cells have very high glycolytic rates that result in the excessive generation of lactate and NADH (Figure 7 and Figure 8) [46]-[48], which are beyond the capacity of pyruvate dehydrogenase and NADH mitochondrial shuttle [47]. The high NADH levels are caused by a disruption of the glycerol 3-phosphate shuttle and a reduction of glutaminolysis as found in breast cancer cell lines (MCF-7 cells) [4]. Therefore, most cancer cells in culture produce a quantity of lactate, implying that the net flow of the intracellular conversion moves from pyruvate to lactate. This movement indicates a switch from mitochondrial oxidative phosphorylation to glycolysis for ATP production.

A previous study by Yin et al., 2004 [21] found that the overexpression of PGC-1 α, peroxisome proliferator-activated receptor gamma coactivator-1 α, decreased cell migration markedly through the upregulation of E-cadherin expression in HepG2 cell lines. Earlier findings by our group demonstrated that CCA cell lines express E-cad and β-catenin in different localization patterns suggesting that alterations of mtDNA and mitochondrial dysfunction might be involved during the malignant transformation of cholangiocytes and hepatocytes [48]. In fact, cadherin-catenin complexes are distorted in numerous cancers such as breast, stomach, colon, and skin cancers. OZ has been described as a metastasizing cell line and, indeed, this cell line demonstrated typical localization patterns of E-cadherin and beta-catenin, both of which are localized in the plasma membrane (data not shown). Among all CCA cell lines studied, HuH-28 is, probably, the most intriguing cell line, as it expresses neither E-cadherin nor plakoglobin, while expresses beta-catenin and N-cadherin with cytoplasmic localization. We also found that cell-cell junction in HuH28 is altered (data not shown). Thus, the investigation of these three cell lines may be considered appropriate, if we keep in mind the degree of invasiveness of the intrahepatic CCA.

This investigation contains some limitations, which may also represent a stimulus for future studies. We believe it is important that a common cell line of exclusively extrahepatic CCA, such as TFK-1, may be included in future investigations and be compared with HepaRG, which is also recognized as a normal hepatocyte cell line retaining p450 activity. In addition, it may also be important to evaluate in detail in all CCA cell lines the genes involved in pathways that may lead to metabolic diseases. As molecular diagnostics begins to embrace next generation technologies, our understanding of the cancer will obviously expand. The extraordinary phenotypic variability of the mitochondrial mutations and polymorphic alterations in the genome of mitochondria generate indeed technical challenges for our understanding of the complexity of carcinogenesis. Our data may open fascinating and exciting fields of research able to discover more singular interaction between metabolism and carcinogenesis, as observed for instance in alpha-1-antitrypsin deficiency, genetic hemochromatosis, and tyrosinemia. Future studies are also required to access the functional role of the various mitochondrial mutations in specific steps of initiation and progression of cancer. Questions remain regarding the progression and functional implications of these mutations in the pathogenesis of CCA. In using animal models, experimental studies targeting the defects of oxidative phosphorylation and their inhibition or reversal, may help to develop strategies able to identify clearly therapeutic protocols for some cancer phenotypes harboring a worse prognosis.

In conclusion, mtDNA alterations are common in cholangiocellular carcinoma. A high copy number of mtDNA mutations suggest that these alterations may contain some promising clinical significance. The molecular mechanisms of the mtDNA changes and mitochondrial OXPHOS defects as well as the intriguing interaction between metabolism and carcinogenesis may provide data for nanotechnology-based mitochondrial-targeted therapies.
